# Melanoma Progression under Obesity: Focus on Adipokines

**DOI:** 10.3390/cancers13092281

**Published:** 2021-05-10

**Authors:** Joanna Olszańska, Katarzyna Pietraszek-Gremplewicz, Dorota Nowak

**Affiliations:** Department of Cell Pathology, Faculty of Biotechnology, University of Wroclaw, Joliot-Curie 14a, 50-383 Wroclaw, Poland; joanna.olszanska2@uwr.edu.pl (J.O.); dorota.nowak@uwr.edu.pl (D.N.)

**Keywords:** adipokines, obesity, melanoma, adipocytes, cytokines

## Abstract

**Simple Summary:**

Obesity is a rapidly growing public health problem and the reason for numerous diseases in the human body, including cancer. This article reviews the current knowledge of the effect of molecules secreted by adipose tissue-adipokines on melanoma progression. We also discuss the role of these factors as markers of incidence, metastasis, and melanoma patient survival. Understanding the functions of adipokines will lead to knowledge of whether and how obesity promotes melanoma growth.

**Abstract:**

Obesity is a growing problem in the world and is one of the risk factors of various cancers. Among these cancers is melanoma, which accounts for the majority of skin tumor deaths. Current studies are looking for a correlation between obesity and melanoma. They suspect that a potential cause of its development is connected to the biology of adipokines, active molecules secreted by adipose tissue. Under physiological conditions, adipokines control many processes, including lipid and glucose homeostasis, insulin sensitivity, angiogenesis, and inflammations. However, when there is an increased amount of fat in the body, their secretion is dysregulated. This article reviews the current knowledge of the effect of adipokines on melanoma growth. This work focuses on the molecular pathways by which adipose tissue secreted molecules modify the angiogenesis, migration, invasion, proliferation, and death of melanoma cells. We also discuss the role of these factors as markers of incidence, metastasis, and melanoma patient survival. Understanding the functions of adipokines will lead to knowledge of whether and how obesity promotes melanoma growth. Further studies may contribute to the innovations of therapies and the use of adipokines as predictive and/or prognostic biomarkers.

## 1. Introduction

Obesity is a rapidly growing public health problem. It is defined as a chronic and severe disorder with an excessive accumulation of fat (adipose tissue), which may lead to various pathologies, particularly cardiovascular diseases, diabetes, musculoskeletal disorders (especially osteoarthritis), and certain types of cancer [1].

Adipose tissue (AT) is the main element of the hypodermis—the deepest layer of the skin [2]. It is mostly composed of adipocytes, as well as stromal cells like pericytes, and endothelial, immune, and pluripotent stem cells. Its role was formerly believed to be limited to energy storage and protection from cold temperature [3]. AT can be classified as white (WAT) or brown adipose tissue (BAT). They differ in morphology, biochemical features, and functions. WAT stores energy reserves as fat, while the function of brown adipose tissue is lipid oxidation to produce heat [4]. In humans, adipose tissue can be located under the skin (subcutaneous fat, SAT) or around internal organs (visceral fat, VAT). VAT, as compared to SAT, contains a higher number of inflammatory and immune cells and a larger percentage of big adipocytes [5]. Furthermore, VAT, in contrast to SAT, showed increased expression of fibroblast growth factor 21 and insulin-like growth factor-binding protein (IGFBP)-5 as well as decreased levels of hepatocyte, insulin-like-1 growth factors, IGFBP-2, IGFBP-3, and IGFBP-6 [6].

Adipocytes, the main cellular component of adipose tissue, are an active source of a wide array of effectors including exosomes, miRNA, lipids, and bioactive molecules called adipokines that may act in a paracrine and endocrine way to control local and systemic metabolic responses [7]. However, the term adipokine is often used in a more general way and refers to molecules synthesized and secreted by whole AT, even if they are produced by other cell types inhabiting this tissue, like infiltrated macrophages or lymphocytes. To the family of adipokines belongs a variety of factors comprising, among others, metabolic markers, inflammatory factors, angiogenic growth factors, and hormones [6]. Under normal conditions, adipokines regulate numerous physiological processes connected to appetite and energy balance. Obesity provokes a complex remodeling of adipose tissue connected to changes in its structure and cellular composition. Excessive adipose tissue accumulation dysregulates the sensitive microenvironment within adipose depots, which consequently alters their physiological processes and leads to adipose tissue-related disorders [8,9]. This multifaceted process includes an increase in adipocyte number (hyperplasia) and size (hypertrophy). The abnormal amount of dysfunctional adipocyte release factors whose function may be associated with different pathologies [10]. Moreover, the latter undergo cell death and contribute to adipose tissue inflammation and macrophage infiltration inducing parallel quantitative and qualitative changes in the cellular composition of adipose tissue. Chronic, low-grade inflammation is a major hallmark of obese adipose tissue [11] and the factors secreted by immune cells should also be considered as molecules contributing to obesity-associated disorders. All this may affect tumorigenesis and be associated with worse outcomes in numerous types of cancer. In addition, cancer survivors with a higher body mass index (BMI) have a higher risk of reoccurrence of this disease [12]. Although the effects of obesity on breast [13], colon [14], and liver [15] cancers have been extensively investigated, the links between obesity and melanoma remain not completely clear and probably multifactorial. Moreover, there exist several controversies about the correlation between melanoma progression and obesity.

Cutaneous melanoma is a cancer derived from melanocytes-melanin-producing cells of the skin. While malignant melanoma accounts for only 4% of all skin cancer cases, it is the cause of the majority of deaths caused by these tumors. Such significant mortality is linked mainly to the heterogeneity and high invasiveness of melanoma [16]. The melanoma niche consists of adjacent cells such as cancer-associated fibroblasts (CAFs), keratinocytes, adipocytes (CAAs), and immune cells, which must also be taken into account as a factor impairing melanoma development and resistance to treatment [17]. The close proximity of adipose tissue and melanoma in the skin would suggest that this tissue may affect the tumor microenvironment and, thus, its progression. However, several reports indicate that there is no convincing association between obesity and risk for malignant melanoma [18,19,20]. On the contrary, other epidemiological studies indicate that there is a strong, positive correlation between BMI and the existence of melanoma in patients [21,22,23,24]. Moreover, diet-induced obesity increased melanoma progression [25,26]; also, enhanced lymphangiogenesis and lymph node metastasis in mice was observed [27].

Obesity-associated remodeling of adipose tissue generates a systemic pro-inflammatory state, in which changes in immune cell populations are observed. Although an increase in the macrophage population is most common, the metabolic state of obese adipose tissue modulates the size of nearly all immune cell populations studied to date [28]. This leads to impaired anti-cancer immune responses in which the tumor-associated cells promote the immune escape of melanoma cells mainly by an imbalanced production of adipocyte-derived factors, which was revised previously [29,30].

**Figure 1:**
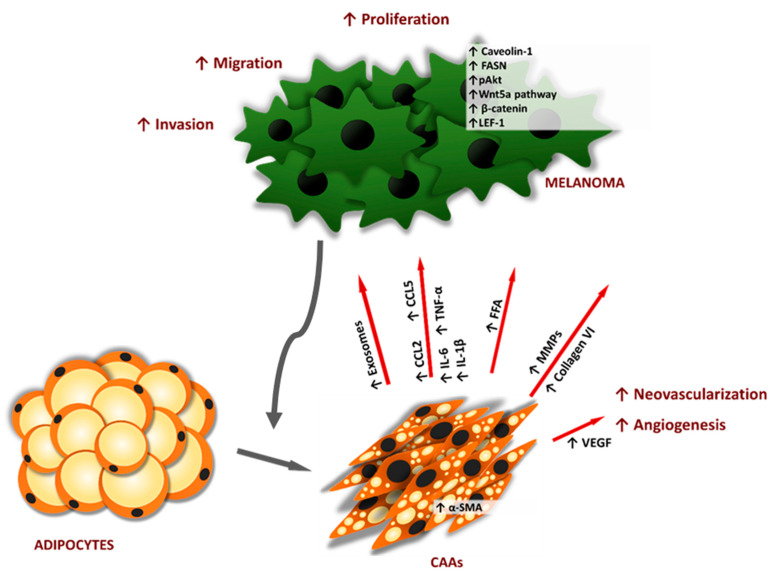
Modification of adipose tissue by melanoma and then influence of modified adipocytes on tumor cells. Adipocytes transformed into CAAs (cancer-associated adipocytes) as a consequence of the tumor neighborhood. They become smaller, have little, dispersed lipid droplets and fibroblast-like phenotypes. They also show abnormalities in the secretion of molecules, which stimulate melanoma tumor progression. Abbreviations: MMPs—matrix metalloproteases, IL-6—interleukin-6, IL-1β—interleukin 1β, TNFα—tumor necrosis factor α, CCL2—chemokine (C-C motif) ligand 2, CCL5—chemokine (C-C motif) ligand 5, FFA—free fatty acid, αSMA-α—smooth muscle actin, FASN—fatty acid synthase, pAKT—phosphorylated protein kinase B, LEF-1—lymphoid enhancer-binding factor 1, VEGF—vascular endothelial growth factor, ↑—overexpression of factors or processes stimulation.

Adipocytes present in the surrounding area of cancer cells, termed cancer-associated adipocytes, display phenotypic and functional alterations. They acquire characteristics different from those of naive adipose tissue cells, like smaller sizes, irregular fibroblast-like shapes, and small lipid droplets as well as an increased level of inflammatory cytokines [31,32]. CAAs secrete collagen VI and matrix metalloproteases (MMPs), which remodel the extracellular matrix (ECM) and promote tumor cell invasion (Figure 1) [33,34]. Moreover, CAAs may undergo conversion to CAFs [35], which further modify the tumor microenvironment and favor melanoma progression that was revised previously [36]. Although most of the research characterizing CAAs comes from studies focused on breast cancer biology, it is now admitted, that when the tumor cells invade the surrounding adipose tissue, adipocytes disappear and fibroblast-like cells accumulate in all tumors growing in an adipose tissue-dominated microenvironment (like gastric, breast, colon, renal, prostate, and ovarian cancers and melanoma) [34]. Co-culture of melanoma cells with adipocytes revealed that cancer cells stimulate a dedifferentiation process of adipocytes toward the fibroblast-like phenotype (higher expression of collagen, MMPs, and α-smooth muscle actin (α-SMA)) (Figure 1) [33]. On the other hand, modified adipocytes promoted melanoma cell migration via activation of the Wnt5a pathway [33].

Adipocytes may support melanoma cell progression in several ways (Figure 1). They can act as a source of nutrients and, thus, have an impact on melanoma cell metabolism. Moreover, the cross-talk between adipocytes and melanoma cells may also occur by adipocyte-derived exosomes, which are then taken up by tumor cells, leading to increased migration and invasion. These aspects of interactions between adipocytes and melanoma were previously revised [17,30].

The cross-talk between the CAAs and cancer cells might be increased in obesity, in which the balance in the secretion of molecules by adipose tissue is altered. Adipokines are the main factors released by adipocytes as well as by the other AT residing cells (like macrophages), which can regulate melanoma cell progression. On the other hand, it was demonstrated that some of them may also be expressed by melanoma cells and serve as markers of melanoma incidences. For this reason, this review will focus on the impact of adipose tissue-derived bioactive molecules, which could affect melanoma advancement under an obesity state. Here, we present how obesity modifies the expression level of factors secreted by adipose tissue. Moreover, we will discuss its influence on melanoma growth, cell migration, and, finally, cancer cell metastasis and summarize which of them may serve as predictive and/or prognostic biomarkers of melanoma progression.

## 2. Leptin

Leptin is a 16 kDa polypeptide firstly isolated as a product of the *ob* gene secreted mainly by WAT [37,38] localized subcutaneously [39]. This hormone can be produced by both preadipocytes and adipocytes [40] as well as other tissues [37]. Leptin’s biological functions were summarized in Table 1, however, its best-known function is suppressing food intake and energy expenditure by acting on the hypothalamus. Moreover, the circulating level of this hormone correlates with the amount of fat [39]. Weight loss after bariatric surgery decreased the level of circulating leptin [41]. It was revealed that the production of leptin by adipose tissue can induce the hepatic expression of C-reactive protein (CRP). Then the CRP binds to leptin and decreases its physiological functions in the central and peripheral nervous system, which may be involved in leptin resistance and obesity [42]. Furthermore, adipokine is currently known for being a pleiotropic hormone. It regulates immune cell functions, induces neoangiogenesis, and regulates vasodilatation and vascular permeability in the adipose tissue [43].

**Figure 2:**
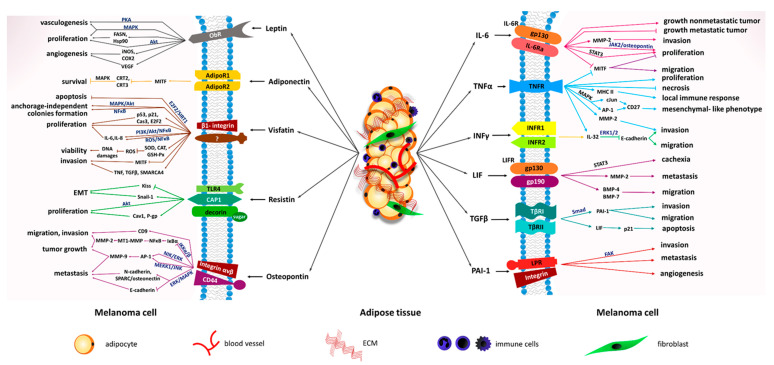
The effect of molecules released by adipose tissue on melanoma cells. Adipocytes secreted factors that interact with their receptors and affect tumor progression via different pathways. Abbreviations: ObR—leptin receptor, AdipoR1—adiponectin receptor 1, AdipoR2—adiponectin receptor 2, TLR4—toll-like receptor 4, CAP1—adenylyl cyclase-associated protein 1, PKA—protein kinase A, MAPK—mitogen-activated protein kinase, FASN—fatty acid synthase, Hsp90—heat shock protein 90, Akt—protein kinase B, iNOS—nitric oxide synthase, COX-2—cyclooxygenase-2, VEGF—vascular endothelial growth factor, CRT2—calreticulin 2, CRT3—calreticulin 3, E2F2—E2F transcription factor 2, SIRT1—sirtuin 1, NFκB—nuclear factor κ-light-chain-enhancer of activated B cells, Cas3—caspase 3, PI3K—phosphoinositide 3-kinase, ROS—reactive oxygen species, IL-6—interleukin-6, IL-8—inter-leukin-8, SOD—superoxide dismutase, CAT—catalase, GSH-Px—glutathione peroxidase, MITF—microphthalmia-associated transcription factor, TNF—tumor necrosis factor, TGFβ—transforming growth factor β, SMARCA4—SWI/SNF-related matrix-associated actin-dependent regulator of chromatin subfamily A member 5, KLF4—Kruppel-like factor 4, NUCB2—nucleobindin 2, Cav1—caveolin 1, P-gp—P-glycoprotein, IKKα/β—nuclear factor kappa—β kinase subunit α/β, IκBα—inhibitor of nuclear factor kappa B, MT1-MMP—membrane type 1-matrix metalloprotease, MMP-2—matrix metalloprotease-2, NIK—nuclear factor inducing kinase, MEKK1—mitogen-activated protein kinase kinase kinase 1, JNK—c-Jun-N-terminal kinase, AP-1—activator protein-1, MMP-9—matrix metalloprotease-9, N-cadherin—neural cadherin, SPARC—secreted protein acidic and rich in cysteine, E-cadherin—epithelial cad-herin, TNFα-tumor necrosis factor α, IFNγ—interferon γ, LIF—leukemia inhibitory factor, PAI-1—plasminogen activator inhibitor-1, IL-6R—Interleukin-6 receptor, TNFR—tumor necrosis factor receptor, INFR1—interferon receptor 1, INFR2—interferon receptor 2, LIFR—leukemia inhibitory factor receptor, TβRI—transforming growth factor β receptor I, TβRII—transforming growth factor β receptor II, LRP—low-density lipoprotein receptor-related protein, JAK2—Janus kinase 2, STAT3—signal transducer and activator of transcription 3, AP-1—activator protein-1, IL-32—interleukin 32, ERK1/2—extracellular signal-regulated kinase 1/2, BMP-4—bone morphogenetic protein 4, BMP-7—bone morphogenetic protein 7, FAK—focal adhesion kinase, ECM—extracellular matrix.

Leptin may act as a pro-tumoral factor in various cancer types [107] and stimulate melanoma growth (Figure 2). While leptin was undetectable in medium from mouse melanoma cells, in vitro studies indicate that it enhances the proliferation of cancer cells [25]. The hormone activated the Akt (protein kinase B) pathway as well as increased the level of fatty acid synthase and heat shock protein 90 leading to the promotion of melanoma cell growth [108]. This adipokine acts in an autocrine manner on melanoma cells which express both ObR (leptin receptor) and leptin. They trigger MAPK (mitogen-activated protein kinase) activation and lead to increased proliferation [25,109]. The promotion of melanoma growth by leptin was also affirmed in research on mice. The peptide probably acts through PKA (protein kinase A) and MAPK pathways, which are conducive to the rise of the NO plasma level and the number of endothelial progenitor cells in peripheral blood leading to vasculogenesis [110].

The autocrine loop of leptin has also been suggested in studies on human nevi and melanomas. Although both types of tissues produce adipokine, ObR was more common in melanoma cells. This allows for autocrine stimulation of tumor growth through pathways dependent on inducible nitric oxide synthase, NO, and COX-2 (cyclooxygenase-2) [109]. Other studies report the existence of a correlation between plasma leptin level and melanoma (Table 2). It was observed that a high level of circulating adipokine increases the risk of cancer but there were no significant differences in comparison to melanoma stages and BMI [111]. In addition, tumor-positive sentinel lymph nodes had enhanced leptin expression as compared to tumor-negative sentinel lymph nodes. This could mean that the hormone can restrict some immunological responses [112]. It was also observed that there was a positive correlation between serum leptin level and metastases to sentinel lymph nodes. Patients had no differences in BMI, so obesity cannot explain the higher levels of the peptide, but perhaps the melanomas themselves are the source of this adipokine. Moreover, hyperinsulinemia and insulin resistance may promote melanoma growth mediated by insulin-like growth factor-I and leptin because patients with diabetes had a worse prognosis [113].

Leptin can display angiogenic (at least partly by vascular endothelial growth factor (VEGF)) and mitogenic properties in the enhancement of tumor growth [139]. On the other hand, the VEGF and ObR expression in mice melanomas was correlated with tumor size but not with the host leptin level. The amount of VEGF in tissue was higher in the melanomas from obese mice and independent of neither circulating VEGF level nor host plasma leptin. Thus, it seems that obesity may promote carcinogenesis regardless of the presence of leptin. However, leptin deficiency significantly attenuates melanoma growth and its high level may accelerate this process [25]. Interestingly, the administration of a low-dose bi-specific nanobody targeting ObR suppressed tumor growth by the inhibition of angiogenesis. It did not affect circulating leptin but increased the level of the soluble form of ObR in the bloodstream and decreased the CD31 expression, which is an endothelial cell marker as well as VEGF receptor. Intraperitoneal injection of this nanobody resulted in a block of leptin signaling in the central nervous system only in high doses [140]. Moreover, the treatment of the A375 melanoma cells with leptin deteriorates the reaction on the chemotherapeutic drug-dacarbazine [108].

## 3. Resistin

Resistin is a circulating hormone firstly identified as being secreted in vivo by WAT [78]. However, it is expressed predominantly in peripheral blood mononuclear cells and macrophages, and minimally in preadipocytes and adipocytes [3,55]. It is currently known that the release of resistin from the adipose tissue of obese patients is caused by a higher level of accumulated mononuclear cells rather than resistin production by adipocytes [102]. An expression of this adipokine is strongly induced by various inflammatory stimuli, e.g., TNF-α (tumor necrosis factor-α), IL-6 (interleukin-6), IL-1β (interleukin-1β), and resistin itself. Its receptor has not been identified, but there are three candidates. They are: toll-like receptor 4 in human myeloid and epithelial cells; adenylyl cyclase-associated protein 1 in human monocytes [80]; and WAT-specific-glycated isoform of decorin [141]. This adipokine was described primarily as affecting glucose metabolism in a manner antagonistic to insulin [78], and mainly links insulin resistance, obesity, and inflammation [142,143]. In addition, a higher level of this hormone in serum, SAT, and VAT was noticed compared to those with a normal weight [56,79].

The serum level of resistin correlates with different types of cancer [144,145]. Regarding melanoma, human research has not yet been conducted. However, the available in vitro studies indicate pro-cancerous activity of resistin in this tumor type (Figure 2). Together with leptin, it increases the proliferation of melanoma cells through the Akt signaling pathway. Moreover, resistin impoverishes the dacarbazine treatment via enhanced caveolin 1 and P-glycoprotein levels [108,146]. This adipokine can participate in the increase of melanoma cells’ aggressiveness by modulation of the epithelial-to-mesenchymal transition (EMT) because it raises the Snail family transcriptional repressor 1 and substantially reduces the metastasis suppressor Kiss1 expression. It was further demonstrated that obese mice unable to produce leptin (ob/ob) had a higher level of resistin in serum together with MCP-1 (CCL-2), IL-6, and TNF-α. Therefore, one can assume that modulation of EMT and a decrease in the metastases suppressor level may promote the aggressiveness of melanoma by obesity [147].

## 4. Visfatin

Visfatin was initially described as nicotinamide phosphoribosyltransferase (NAMPT) and pre-B cell colony-enhancing factor. The enzyme catalyzes the conversion of nicotinamide to nicotinamide mononucleotide (NMN) in nicotinamide adenine dinucleotide (NAD) synthesis [148,149]. However, it is known today that they are terms for the same molecule, which is a 52 kDa protein [3]. Visfatin is expressed mostly in VAT [103,104], mainly by macrophages infiltrating the white type of this tissue [102], but also in lymphocytes, bone marrow, skeletal muscle, hepatocytes, and cardiomyocytes [3,148,150]. In addition to its role in the regulation of cellular energetics, visfatin is best known as an insulin mimetic factor [148] as well as a proinflammatory adipokine. Additionally, visfatin may cause angiogenesis and endothelium dysfunctions because it increases the expression, protein level, and activation of VEGF, metalloproteases (MMP-2 and MMP-9), adhesion molecules (ICAM-1, VCAM-1, and E-selectin), and inflammatory cytokines (IL-6 and IL-8) [151,152].

Visfatin is positively associated with obesity (Table 1) and malignancies [153,154,155,156,157]. In human melanoma cells, the level of this hormone was higher in comparison to melanocytes [115]. Its transcription is controlled by BRAF/MEK (mitogen-activated protein kinase)/ERK (extracellular signal-regulated kinase) cascade following activation of STAT5 (signal transducer and activator of transcription 5) [158]. The transcription factors NF-κB (nuclear factor κ-light-chain-enhancer of activated B cells), STAT3, and HIF-1α (hypoxia-inducible factor 1α) regulate the increased expression of NAMPT [117]. It was observed that recombinant visfatin stimulates melanoma cell proliferation in a dose-dependent manner, but the mechanism of this process is not fully understood. This adipokine can reduce DNA damage through the enhancement of antioxidant enzyme activity (such as superoxide dismutase isoenzymes, catalase, and glutathione peroxidase) in melanoma cells and, thus, promote its viability. On the other hand, an increase in proliferation may be the result of enhanced production of IL-6 and IL-8 via the PI3K (phosphoinositide 3-kinase)/Akt/NFκB pathway or/and reactive oxygen species (ROS). ROS, which causes oxidative stress, may activate NFκB itself, leading to the production of larger amounts of pro-inflammatory cytokines (Figure 2) [159,160].

Visfatin may redirect melanoma cells to a more invasive phenotype, which is involved in targeting therapy resistance. As the transcriptomic analysis revealed, overexpression of NAMPT is associated with downregulated expression of genes committed to proliferation and enhanced genes involved in the invasion. Moreover, visfatin-activated genes are associated with cell movement and migration (e.g., *TNF*, *TGFB*, *SMARCA4*, *ZEB1*) [158].

In around half of all melanoma cases, a mutation in the BRAF (most often V600E) is detected [29]. This leads to constitutive activation of the BRAF-MEK-ERK axis and MAPK signaling [161]. Based on this notice, BRAF and MEK inhibitors were approved for clinical use. Unfortunately, melanoma patients often acquire resistance to this form of therapy [29]. Moreover, visfatin may take part in the emergence of resistance to BRAF inhibitors by melanoma cells because the NAD level was rising during resistance development. Moreover, melanoma cells resistant to BRAF inhibitors (BRAFi) were unusually sensitive to inhibitors targeting NAMPT, which caused decreased cellular NAD levels. The cited study also proved that the adipokine is committed to determining the aggressive behavior of BRAF-mutated melanomas. Melanoma cell lines with visfatin overexpressing advanced the resistance to BRAFi earlier, and they also grew faster and more effectively healed wounded areas [117].

NAMPT also plays a role in apoptosis resistance through NAMPT/E2F2 (E2F transcription factor 2)/SIRT1 (sirtuin 1) pathway [115]. Silenced visfatin gene using siRNAs or inhibited by FK866 (a highly specific noncompetitive inhibitor of NAMPT) decreased the NAD+ level, which led to enhanced melanoma cell death [158]. Other data shows that besides the pro-apoptotic effect, the FK866 or silencing NAMPT gene act in an anti-proliferative way in melanoma cells through activation of p53, p21, and Caspase-3 as well as a raised level of E2F2 [115]. Moreover, treatment of BRAFi melanoma cells with FK866 contributes to a change in mitochondrial morphology, loss of membrane potential, and accumulation of ROS, which disturbed energy production. These cells were arrested in the S or G2/M phases of the cell cycle because of downregulated B1/Cdk1 cyclins and were directed to the mitochondrial-dependent apoptotic pathway. Decreased levels of Mcl-1 and Xiap (pro-survival molecules) and increased Bax and activated Caspase3 levels were observed. Administration of very high concentrations of nicotinic acid was not able to completely rescue cells blocked in the G2/M phase, in contrast to S cells [117]. On the other hand, in another study, enzymatic inhibition of NAMPT did not result in lower melanoma cell viability [114].

Interestingly, melanoma actively releases eNAMPT (extracellular NAMPT), and cells resistant to BRAF inhibitors have higher eNAMPT levels [118]. This form of the enzyme could act in an autocrine and paracrine manner on mouse and human melanoma cells, leading to the activation of various signaling pathways. It triggers MAPK, Akt, and NFκB expression, which does not contribute to increased proliferation but enhances anchorage-independent colony formations by melanoma cell lines [150]. Studies in mice also focused on the silencing or inhibition of visfatin. It was observed that mice with melanoma development from BRAFi cells were characterized by higher eNAMPT levels in plasma [118]. Treating the melanoma with eNAMPT targeted shRNA led to a slowdown of tumor growth [150]. Moreover, mice with subcutaneously injected melanoma cells administrated with the combination of FK866 and BRAFi survived longer than those treated with one substance did. This mix was not toxic because BRAFi cells use primarily NAMPT to NAD synthesis, whereas normal cells employ various other pathways. On this basis, it is concluded that the combination of NAMPT and BRAF inhibitors has the potential to be a new therapy for BRAFi-sensitive patients with melanoma [117].

The visfatin gene can also be helpful in the diagnosis of melanoma (Table 2). It was found among the genes, whose expression enabled the separation of melanoma from atypical nevi or normal skin [116]. Other research confirmed a higher expression of NAMPT in melanoma in comparison to melanocytes, as well as in biopsies from patients with BRAFi as compared to before the acquisition of resistance [117]. The visfatin level can be helpful in diagnosis based on immunohistochemical staining. The overexpression of NAMPT was observed in the vertical growth phase and in melanoma metastases when compared to melanocytes [114]. Furthermore, NAMPT expression in the tumor was increased in people who died from melanoma and in the advanced stages of this cancer. Patients with high mRNA NAMPT levels lived shorter lives compared to those in low NAMPT expression groups. Thus, visfatin can be a potential prognostic marker in melanoma [115]. Additionally, it can act in the prognosis of the tumor mass and therapy response. Patients with BRAF-mutated melanoma metastases had enhanced eNAMPT levels in plasma as compared to patients without them and to healthy controls. The researchers observed a direct correlation between eNAMPT plasma levels and markers of the tumor mass. The extracellular visfatin level decreased the response to therapy with BRAF/MEK inhibitors but increased again upon melanoma progression and development of BRAFi resistance. It was shown that a high eNAMPT level is associated with a shorter overall survival of patients [118].

## 5. Osteopontin

Osteopontin (OPN) was known primarily as phosphorylated secretory protein, sialoprotein released from bones. Nevertheless, later, its intracellular form was also described [50,162]. Humans can express five isoforms of this protein due to alternative splicing of a single mRNA transcript [162]. However, the differences in its molecular weight (41–75 kDa) are explained by various posttranslational modifications [45]. Under physiological conditions, the hormone is expressed in various cells, including adipocytes, and is released to different body fluids [45,47,163]. Osteopontin signaling is mediated by its receptors: some integrins αvβ and CD44v3, v6-10 variants [52,162]. The wide expression of OPN proves its involvement in various physiological and pathological processes (Table 1) [49]. Osteopontin promotes angiogenesis [51] and neovascularization [52]. Moreover, it plays a role in the development of insulin resistance (through increasing accumulation of macrophages in adipose tissue), and cancers [50]. The level of this adipokine was higher in plasma, peripheral blood mononuclear cells, and AT of obese and overweight patients compared to lean individuals. In addition, its concentration was correlated with body fat percentage [47,48].

OPN and its receptors are involved in the pathology of various cancers, which are associated with the angiogenesis, proliferation, migration, invasion, and metastasis of cells. Its expression is also connected to resistance to therapy and poor prognosis [50,51,164]. Melanoma in vitro studies indicate that the osteopontin gene expression is higher in tumor cells than in melanocytes. The hormone was present in melanoma cell lines in contrast to melanocytes [120]. OPN was released as a soluble protein from cell lines generated from metastases of freshly explanted melanomas [121]. Moreover, the OPN mRNA expression was higher in the metastatic uveal melanoma cell line (MUM2B) as compared to the aggressive primary uveal melanoma cell line (M619) and nonaggressive (OCM1a); also M619 had an increased level compared to OCM1a [128]. It was demonstrated that the hormone may take part in the regulation of melanoma proliferation because the blocking of OPN expression by siRNA decreased the growth rate of cancer cells [120]. On the other hand, an inconsistent result was obtained by another group, which concluded that osteopontin was not involved in proliferation, but had a positive effect on the adhesion of mouse malignant melanoma cells (B16) [165].

Osteopontin may promote the migratory and invasive ability of melanoma through integrin αvβ3 activation and decreased expression of tetraspanin CD9 [124]. By the interaction with αvβ3, this hormone activates the pro-MMP-9 via NIK (nuclear factor inducing kinase)/ERK and MEKK1 (mitogen-activated protein kinase kinase kinase 1)/JNK (c-Jun N-terminal kinase) mediated pathways [125]. The other mechanism is based on the involvement of OPN in the NFκB translocation to the nucleus and results in increased MT1-MMP (membrane type 1-matrix metalloprotease) expression. Then it causes an elevated level of MT1-MMP on the cell surface, which activates pro-MMP-2 (Figure 2) [127]. In addition, melanoma cell migration may be increased by the ERK/MAPK pathway [166]. To the pathological function of the hormone belongs participation in the regulation of EMT. The expression of osteopontin, N-cadherin (neural cadherin), and secreted protein acidic and rich in cysteine/osteonectin (proteins belonging to the EMT group) was associated with the increased frequency of melanoma metastasis. An enhanced N-cadherin level was accompanied by the loss of epithelial cadherin (E-cadherin) expression [167].

OPN can also promote macrophage-mediated migration and angiogenesis of melanoma because it enhances TAM (tumor-associated macrophage) activation and tumor infiltration. The protein binds to α9 integrin, which activates p38 and ERK signaling pathways and, finally, increases the COX-2 and prostaglandin 2 (PGE2) expression in these macrophages (Figure 2). Therefore, OPN-activated TAMs had higher MMP-9 levels and were able to promote melanoma growth [168].

Osteopontin stimulated melanoma cell growth and metastasis to the lung mediated by NIK and MEKK1 signaling [125]. Tumor development from cells previously treated with the adipokine was larger and produced more pro- and active MMP-2 in comparison to the control [127]. Mice injected with melanoma cells previously treated with OPN were characterized by enhanced lung metastases [125]. Oppositely, in osteopontin-deficient mice, a reduction was observed in the number of bone and lung melanoma metastases [165]. OPN deficiency inhibited the melanoma mitotic features and angiogenesis. Mice without osteopontin had suppressed the amount of COX-2 positive TAMs in melanoma tumor and PGE2 serum level. Conversely, human melanoma biopsies had enhanced macrophage infiltration, mainly OPN-positive and COX-2 positive TAMs, which was correlated with increased tumor growth and angiogenesis [168].

The level of OPN in formalin fixed melanoma tissue was correlated with the changed expression of 32 genes involved in proliferation, cell division, interaction between tumor cells and matrix, DNA repair, replication, cell cycle, cell motility, and signaling [126]. It is currently suggested that osteopontin may serve as a melanoma metastasis marker as well as a prognostic biomarker for both the survival of metastatic-free patients and overall survival [123,126]. This adipokine was overexpressed in metastatic melanoma and increased during the primary cancer thickening [121]. The expression of osteopontin was lower in benign nevi, higher in thin melanoma, and the largest in thick primary melanomas. In all tested samples an inverse correlation between OPN and CD9 expression was observed [124]. The overexpression of OPN and pNIK was also noticed in human malignant melanoma biopsies. This was correlated with the severity of the disease according to Clark level, Breslow thickness, and expressed tumor grade [125]. The increased level of OPN in invasive and metastatic melanoma may suggest that its overexpression is acquired during the first stage of invasion [120]. Besides association with an increase in tumor thickness and Clark level, it is correlated with mitotic index (which means the number of mitoses per mm2) and decreased patient survival. A high level of OPN carried worse clinical outcomes in people with primary melanoma [122,123]. Immunohistochemistry staining showed diffuse osteopontin expression in a hepatic tissue specimen of uveal melanoma metastases and increased serum level specifically correlated with liver metastases [128]. On the other hand, research was conducted that did not show differences between OPN levels in invasive primary or metastatic melanomas. Primary melanoma patients did not show associations between OPN expression and lymph node invasion, tumor subtype, its site, as well as five-year patient survival [120]. Potential role of osteopontin as a biomarker was summarize in Table 2.

## 6. Adiponectin

Adiponectin, also known as Acrp30, adipoQ, or GBP28, has opposing actions to leptin. This 30 kDa adipokine [58] is expressed by mature AT and secreted into serum [55]. Though its main sources are mature adipocytes of visceral WAT [53], adipokine is also expressed in other, different tissues [62,169]. Adiponectin sensitizes to insulin [169] and in skeletal muscle enhances glucose uptake and fatty acid oxidation. This protein works as a proangiogenic and antiapoptotic factor in endothelial cells. Furthermore, it is an anti-inflammatory factor preventing neutrophil apoptosis [170]. This hormone stimulates the storage functions of AT because it helps in adipocyte differentiation, promotes adipogenesis, and enhances the accumulation of triglycerides [169]. Additionally, this protein negatively regulates CRP and TNF expression in adipose tissue [171]. Interestingly, it is abundantly present in serum and its levels are decreased in various pathological conditions including obesity [169]. In obesity, the serum adiponectin concentration and SAT level were lower than they were in people of normal weight [56]. Adiponectin is considered to be an anti-tumor factor, which acts via the suppression of proliferation, migration and by promoting apoptosis. The peptide and its receptor levels are decreased in various cancers [62,172].

Adiponectin participates in the inhibition of melanin synthesis in melanocytes. It activates the AMPK (5′ AMP-activated protein kinase) pathways leading to inhibition of the transcription activity of CREB regulated transcription coactivator 2 and 3. This results in the decrease of MITF (microphthalmia-associated transcription factor) expression, which plays a role in melanocyte differentiation and melanoma cell survival (Figure 2) [173,174]. Moreover, adiponectin receptors may be involved in tumor development. It was observed that AdipoR1 overexpression is common in obesity-associated cancers. Melanoma cells as well as melanocytes express both AdipoR1 and AdipoR2 [173,175].

Adiponectin knockout mice characterized enhanced growth of subcutaneously injected melanoma cells, but depletion of this adipokine in the tumor niche did not affect apoptosis, angiogenesis, and mitosis of cancer cells. The reason for the increased tumor growth may be the reduction of macrophage infiltration observed in the study [176].

Clinical data indicate that the serum adiponectin level was lower in patients with uveal melanoma and choroidal nevus compared to healthy individuals. It was also decreased in a group with metastases as compared to nonmetastatic patients. Thus, the low concentration of circulating hormone and additional insulin resistance may promote melanoma growth and increase its aggressiveness [129]. Similar results were obtained in uveal melanoma with monosomy-3 research, in which the deficiency of adiponectin and insulin resistance were associated with increased tumor metastasis [130].

## 7. Nesfatin-1

Nesfatin-1 was firstly detected as being secreted from the hypothalamic nuclei as a factor responsible for controlling appetite [78]. Nesfatin-1 is an 82-amino acid derivative of the N-terminal of nucleobindin 2 (NUCB2), which is also a protein precursor of nesfatin-2 and -3, two peptides with so-far unknown functions [64]. Nesfatin-1 administration not only is limited to the central nervous system but is also present in several peripheral tissues, and in adipose depots in both SAT and VAT. The polymorphisms [177] or mutations [178] of the nesfatin gene, NUCB2, might be associated with the development of obesity. Recently, an increasing number of studies suggest that nesfatin-1 can be a prognostic factor in several types of cancer, like endometrial [179], gastric [180], and bladder cancer [181]. Moreover, NUCB2 may act as a promoter of tumorigenesis and metastasis in breast cancer and renal cell carcinoma [182,183].

Adaptation to endoplasmic reticulum (ER) stress by melanoma cells may be one of the reasons for its resistance to therapy. Zhang et al. demonstrated, that KLF4, a zinc finger-type transcription factor, was stimulated by ER stress supporting melanoma cell metastasis. Interestingly, KLF4 was shown to regulate transcription of *NUCB2* by binding to its promoter which induced melanoma ER stress resistance, tumor growth, and cell metastasis in vitro and in vivo. Moreover, a higher level of KLF4 correlates with elevated NUCB2 in human melanoma tissues, suggesting that *NUCB2* may be important in the regulation of melanoma metastasis under ER stress [184].

## 8. Chemerin

Chemerin, which is also known as the retinoic acid receptor responder 2 (RARRES2), is an adipokine expressed in adipose tissue and the liver. This protein is a ligand for chemokine-like receptor 1 (CMKLR1), G-protein coupled receptor 1 (GPR1), and C-C chemokine receptor-like 2 (CCRL2), which is expressed mainly in dendritic cells, macrophages, and some adipocytes [185]. WAT expresses high levels of chemerin and CMKLR1, and the autocrine/paracrine signaling of this adipokine is suggested. However, both mentioned proteins are also present in brown adipose tissue [67]. The majority of serum chemerin is biologically inactive. Only after chemerin proteolytic processing at its C-terminal by different proteases, are diverse isoforms generated that vary in their activity, what was previously revised [186]. This molecule is considered a multifaceted adipokine, as it is involved in the regulation of multiple processes presented in Table 1 [69]. A positive correlation between chemerin level in serum and obesity-related features, such as insulin resistance, body mass index, and serum triglycerides, suggests a function of this adipokine in metabolic diseases [187].

Chemerin’s role in cancer progression is still not established, as it can act in both anti-tumoral and tumor-promoting ways, which is mediated by different mechanisms, like the stimulation of angiogenesis or the recruiting of innate immune defenses [185]. Its expression is down-regulated in the majority of human tumors, including melanoma. This down-regulation may be connected to malignant transformation of cells, as the chemerin level was also diminished in melanoma cells in comparison to primary melanocytes. Overexpression of chemerin in murine melanoma cells inhibited melanoma growth in vivo, but not in vitro. Reduced tumor formation in mice was connected to an altered profile of tumor-infiltrating cells and recruitment of NK (natural killer) cells in a CMKLR1-dependent way [131]. Chemerin-deficient mice had accelerated tumor growth and impaired recruitment of tumor-infiltrating NK cells that express CMKLR1 [188]. Interestingly, injection of this adipokine had a similar effect as its overexpression in melanoma cells [131], suggesting that exogenous chemerin present within the tumor niche due to expression by stromal or tumor cells can stimulate host immune defenses to inhibit cancer progression.

## 9. Apelin

Apelin is an endogenous peptide identified as a ligand of the G protein-coupled receptor APJ. It is secreted as a 77-amino acid prepropeptide, which is next cleaved from the C-terminus, to produce a family of apelin peptides: apelin-36 or shorter apelin-17, -12, and -13, which also exists as a pyroglutamyl form [Pyr1]apelin-13 [189]. This adipokine is produced in mature adipocytes and other compounds of WAT [70]. Moreover, in mice, its transcript was also detected in brown fat [77]. Under normal conditions, apelin and its receptor are involved in the regulation of many physiological processes, such as body fluid homeostasis, the regulation of the cardiovascular system, angiogenesis, and energy metabolism. Recently, many studies focused on the participation of apelin in pathological processes, including heart failure, respiratory diseases, and diabetes [75,190]. This adipokine is also involved in obesity, in which case its level is higher in serum and adipose tissue [62,72]. Moreover, apelin peptides are considered to be factors that stimulate tumor growth in several types of cancer, including gastric [191], ovarian [192], colon [193,194], hepatocellular [195,196], and oral squamous cell carcinoma [197]. By regulation of processes connected to cancer invasiveness, like cell migration, apoptosis, or angiogenesis, apelin may participate in the induction of metastasis [75,198].

Apelin can also promote melanoma progression. Mouse melanoma cells stably transfected with this peptide implanted into mice formed bigger tumors in comparison to control cells. The overexpression of apelin not only stimulated tumor growth but also led to increased intratumoral lymphangiogenesis. Moreover, Berta et al. observed enhanced lymph node metastasis in mice carrying apelin-transfected primary tumors [199]. Next, research revealed that, also, apelin overexpression increased the number and size of melanoma lung metastases in mice. Additionally, in vitro studies showed that higher apelin expression enhances the migratory and invasive abilities of melanoma cell lines, but does not influence their proliferation [132]. However, the inhibition of miR-4286 altered the mRNA expression of, among others, this hormone gene that next was implicated in the regulation of melanoma cell proliferation and apoptosis [200]. Interestingly, apelin and VEGF plasma concentrations were more elevated in patients with melanoma than in healthy individuals [132].

## 10. Chemokines

Chemokines (or chemotactic cytokines) are low molecular weight (8–13 kD), heparin-binding proteins, and chemotactic factors that are released from various cell types [201]. They are active as monomers but can form homo- or heterodimers, as well as higher-order aggregates [202]. Due to their structure, chemokines are categorized into four families: CC, CXC, (X)C, and CX3C. Based on function, they can be divided into inflammatory, homeostatic, and dual-function chemokines [201]. In general, homeostatic chemokines are constitutively expressed in lymphoid and other organs. They are essential for tissues and organs as the mediators of homing mainly lymphocytes in preparation for immune responses triggered by injuries [203]. In contrast, inflammatory chemokines are produced by circulating leukocytes and other cells only upon activation. They attract leukocytes to inflamed tissues and their expression is frequently triggered by pro-inflammatory factors (TNF, IFN-γ) and bacterial products, e.g., lipopolysaccharide (LPS). This subgroup includes CCL1–5 and CXCL1–11 molecules [204].

One of the most utilized chemokines is CCL5 (RANTES). This factor is expressed beyond the immune and epithelial cells [82] also in adipocytes of WAT [70]. Mainly in subcutaneous fat after UV stimulation, where it can decrease expression of lipogenic enzymes and sterol regulatory element-binding protein-1, which impair triglyceride synthesis [205,206]. Interestingly, adipocytes also express other chemokines such as CCL2 and CXCL8, as well as their receptors including CXCR1, CXCR2, CCR1, CCR4, CCR5, and CCR10 [207]. Increased levels were observed of some chemokines and their receptors in visceral and subcutaneous AT specimens from obese individuals [71,72,73,208]. Additionally, overweight patients had upregulated CCL2, CCL3, and CCL5 concentrations in serum [71,73].

Chemokines and their receptors can be expressed by various cancers and take part in the angiogenesis, proliferation, and metastasis of tumor cells [204]. Melanoma cell lines overexpress CCL5 and have decreased levels of CCR1, CCR2, and CCR3. Cells with higher invasiveness have enhanced expression of CCL5 as compared to less aggressive cells [133,209]. Moreover, the CCR3 was overexpressed in biopsies from patients with malignant melanoma. This may indicate that the receptor can increase the tumor metastatic potential [210].

## 11. Interleukins

### 11.1. Interleukin-32

Interleukin-32 was identified primarily as NK cell transcript 4 (NK4) expressed in T lymphocytes and NK cells [83]. It exists in nine splice variants, formed as a result of the alternative splicing of eight exons [211]. All isoforms are biologically active but IL-32γ is the most active [212]. Except for T and NK cells, other immunological (like macrophages and monocytes), non-immune cells, and tissues produce IL-32 [83,211]. It was also observed that the level of this peptide is increased in the serum and AT of obese patients [83,85]. Moreover, the concentration of this interleukin correlates with BMI, waist circumferences, and waist-to-hip ratio. IL-32 is involved in inflammation because it enhances the expression of some inflammatory markers. It also increases the transcription of HIF1A, VEGFA, MMP-9, and OPN, which is conducive to ECM remodeling in adipose tissue [85]. The cytokine is expressed in various cancer cells, where it promotes metastasis, migration, invasion, and also apoptosis [83,84].

Furthermore, IL-32 was detected in most melanoma cell lines. The exposition of non-producing IL-32 cells to TNF-α and IFNγ induced the expression of this interleukin by increasing the activity of the promoter. Its expression by dedifferentiated melanomas can be connected to the initiation of a pro-inflammatory tumor microenvironment and associated with a more invasive (with a lower level of E-cadherin) phenotype of cancer cells (Figure 2) [134]. Moreover, melanoma cells with higher migratory abilities possess increased IL-32α expression. This factor acts via activation of the ERK1/2 pathway, which results in the inhibition of E-cadherin expression and enhancement of actin polymerization. In vivo research shows that it increases lung metastasis in mice with melanoma, so IL-32 also acts as a pro-invasive molecule [213].

### 11.2. Interleukin-6

Interleukin-6 is a glycosylated [89] secretory protein with a molecular mass of 26 kDa [87]. It is synthesized by various normal and cancer cells [86]. The sources of the cytokine are subcutaneous and visceral AT, especially adipose tissue macrophages (ATMs) [124]. The level of IL-6 in serum was increased in obese patients compared to normal-weight individuals [71]. Its biological functions include stimulation of the differentiation of B lymphocytes into plasma cells, induction of IL-2 production, and enhancement of endothelial growth factor production [88]. It is also a key factor in the control of Th17/Treg (regulatory T cells) balance [89]. Furthermore, the cytokine takes part in the progression of several diseases including various cancer types [214].

A relatively long time ago, an increased level of IL-6 in serum was observed in patients with melanoma. Thus was proposed its role as a potential prognostic biomarker [136]. It was also found that this interleukin suppresses melanoma cell line growth and cells isolated from nonmetastatic cancer, but stimulates metastatic melanoma cells [88]. However, the mechanism of action of this molecule is not fully understood. Currently, an association is found between adipocytes and melanoma (Figure 2). Tumor cells co-cultured with adipocytes characterized a switched phenotype from proliferative to more invasive. This was due to the release of the soluble factors: IL-6 and TNF-α by adipocytes, which reduced the MITF abundance. This, in turn, decreased the expression of miR-211 involved in melanoma phenotypic plasticity. Additionally, the inhibition of miR-211 boosted the TGF-β (transforming growth factor β) receptor levels leading to sensitization of cancer cells to environmental TGF-β. Finally, it increased its signalization and, in that way, reversibly enhanced invasion ability [215]. Moreover, IL-6 and osteopontin upregulated the transcript levels of chemokines (CXCL1, CXCL2, and CXCL5) [216].

The increased amount of adipocytes in the bone marrow of obese mice was promoted through IL-6/JAK2/osteopontin axis melanoma growth in this tissue. This suggests the existence of the feedback loop of OPN and IL-6 in which the hormone acts both on melanoma, stimulating its proliferation, and on adipocytes, enhancing IL-6 and TNF-α synthesis [216]. Moreover, the interleukin acts in an autocrine or paracrine manner and increases cell proliferation followed by activation of the STAT3 pathway [217].

The association between melanoma and AT was also proved in situ. In the tumor specimens in which adipose cells were in proximity to melanoma, there was an increased number of cancer nests. In higher cancer stages, melanoma cells were closer to the adipocyte cells than in lower stages, which may indicate faster invasive progression. Furthermore, only melanoma samples from a location near adipocytes had IL-6 expression in the upper dermal regions [215]. IL-6, CRP, and the neutrophil/lymphocyte ratio can be prognostic markers of melanoma. Metastatic melanoma patients treated with chemotherapy or immune checkpoint inhibitors with increased levels of the aforementioned factors characterized shorter overall survival [218]; also, patients with melanoma overexpressing IL-6R had shorter survival [215].

### 11.3. Leukemia Inhibitory Factor

Another cytokine, which belongs to the IL-6-type cytokine family, is leukemia inhibitory factor (LIF) [87]. Its mass (37–62 kDa) depends on the degree of glycosylation. This factor is released by developing embryos, various adult organs, and different cells including adipocytes [6] and macrophages [92]. It exerts a pleiotropic effect on cells depending on the type of cell and signaling pathway [219]. The LIF functions, like other IL-6s, include B-lymphocyte stimulation, balance regulation between Treg and effector T cells, as well as metabolic functions [220]. Moreover, LIF takes part in adipocyte differentiation through LIF receptor-gp130 signaling [91]. However, LIF is probably a negative regulator during the adipogenous differentiation of human bone marrow mesenchymal stem cells [220]. It was observed that hypothalamic inhibition of this factor in obesity-resistant mice switched them into the obesity-prone phenotype, which indicates that this adipokine is an obesity-protectant [221]. On the other hand, a high-fat diet decreased the LIF gene transcription in the brain stems of rats which may contribute to permissive overconsumption and the development of diet-induced obesity [93]. Moreover, it plays a role in the pathology of various diseases including cancers [222]. In many tumors, it is overexpressed and correlated with poor prognosis. It contributes to the proliferation, metastasis, and therapeutic resistance of cells. The protein is released into serum, which makes it a potential cancer biomarker. On the other hand, LIF suppresses tumorigenesis in leukemia, medullary thyroid carcinoma, and gastric and cervical cancer [94].

LIF is also produced in melanoma cells [137] and is overexpressed in comparison to melanocytes. Moreover, hypoxia, especially HIF-1α [223] and TGF-β, increased its production [224]. Treatment of melanocytes with recombinant LIF leads to stimulation of their migration and invasion. Interestingly, this study did not reveal activation of the STAT3 pathway typical of the IL-6 family. However, decreased expression of LIF reduced bone morphogenetic proteins 4 and 7, which may in part be because of involvement in bone metastasis or melanoma-induced bone destruction [223]. STAT 3 activation has been reported in another study. It has been shown in vitro that the reduction effect of TGF-β on melanoma growth was mediated by LIF. In addition, the TGFβ/LIF/p21 axis caused melanoma cell cycle arrest in the G1 phase and cell death mediated by caspase. LIF knockdown blocked STAT3 phosphorylation by TGF-β, indicating that this factor is involved in response to LIF [224]. Both the above-cited research studies concluded LIF’s influence on early melanoma stages. On the other hand, there is a relatively distant study indicating no effect of LIF and IL-11 on the stimulation of melanoma cell line growth [225].

LIF receptor (LIFR) is also involved in the progression of melanoma. In vitro studies indicated increased expression of LIFR in melanoma cells as compared to melanocytes. The knockdown of its gene led to decreased migration through STAT3 and partly by p53, reducing MMP2 activation (Figure 2). This was confirmed by in situ studies, in which a positive correlation between LIFR expression and malignant melanocytic lesion, metastasis, tumor thickness, and five-year survival of patients was observed [226].

Furthermore, mice research provided evidence that LIF regulated the development of cachexia in melanoma [227] and bone metastasis probably through stimulation of osteoclastogenesis. Animals with LIF silencing had fewer osteoclasts in the resorption pits of the bone than parental and control. In addition, they were characterized by a decreased number of incidences of bone metastases and tumor colonies and longer periods between the inoculation of the melanoma and the first detection of metastases [137].

## 12. Tumor Necrosis Factor

The tumor necrosis factor superfamily contains 19 members, which are mostly expressed by immune cells, like TNF-β (known also as LTα) produced in NK, T, and B cells; however, the generating of TNF-α is more diverse [96,228]. It was observed that TNF-α is released by AT, but is produced by the non-fat cells present in the tissue rather than adipocytes [95], primarily by ATMs [86]. The level of TNF-α in adipose tissue was inversely related to that of adiponectin [95] and was increased in obese individuals [72]. Some ligands of the TNF family (e.g., TNF-α) are presented in transmembrane and soluble forms, whereas others (such as TNF-β) are presented only as a soluble protein. Therefore, TNF-α can do a “reverse signaling” that transmits a signal from the receptor to the cell with the transmembrane form of the ligand [98]. Currently, 29 receptors (TNFR) that mediate in the function of TNF family members are identified [96]. They regulate various physiological and pathological functions. TNF-α can induce lipolysis, which may cause a sustained inflammation and tissue insulin resistance; it is also associated with autoimmunological diseases. Interestingly, in cancer, it acts as a pro- and anti-tumor factor [96,229].

TNF function in melanoma is not fully clear. Cancer cells release large amounts of cytokines including TNF-α, IL-6, IL-12, VEGF, and TNFR2, which enhances MMP-2 enzymatic activity and leads to increased invasiveness of melanoma. Additionally, in a more aggressive melanoma cell line, higher levels of IL-6, eotaxin, and TNF-α were detected [133]. However, other studies indicate that only cells with N-RAS mutation (changing Gln to Arg, which occurs in 15–20% of melanomas [6]) constitutively express and release IL-1α, IL-6, and TNF-α into the medium [135].

A low level of TNF-α in mice melanoma cells caused higher cancer progression by decreased necrosis, increased proliferation rate, and local microvascular density as opposed to enhanced amounts of the cytokine [230]. TNF was also involved in de novo expression of MHC class II in melanomas, which results in favoring the accumulation of tumor-specific CD4+ T cells in the tumor microenvironment and local immune response. Increased immunosuppressive capacity was also due to TNF-induced reduction of the immune sensitivity of cancer in the IFNγ-rich tumor niche (Figure 2) [231]. Moreover, this factor was able to induce CD73 expression via MAPK signaling, leading to dedifferentiation and therapy resistance in melanoma cells [232]. On the other hand, TNF may be involved in the immune escape of melanoma by the reduction of tumor-infiltrating CD8+ T cells, which is mediated by TNFR1 [233].

## 13. Plasminogen Activator Inhibitor-1

Plasminogen activator inhibitor-1 (PAI-1) is a 47 kDa glycoprotein that belongs to the serpin family E1. It is a key inhibitor of tissue (tPA) and urokinase plasminogen activator (uPA). This molecule is expressed in a majority of tissues, and those rich in blood vessels usually generate higher levels of this cytokine [86]. It is produced in ATMs and adipocytes of subcutaneous and visceral WAT [70,72,86]. The expression of PAI-1 is increased by several transcriptional factors and in the inflammation by some cytokines, LPS, angiotensin II, insulin, and hypoxia [86,101]. The primary function of PAI-1 is the suppression of fibrinolysis by inhibiting the conversion of plasminogen into active plasmin. Then this protein is unable to activate MMP-2, MMP-3, and MMP-9 [234] mediated by PA [86]. Additionally, PAI-1 is involved in cell motility, by interactions with some extracellular matrix components [101]. Besides this, it plays other physiological and pathological roles [235]. This molecule is involved in glucose and lipid metabolism [86]. Moreover, its serum and adipose tissue levels are higher in obese people [72,99], due to an increase in the AT and enhanced PAI-1 production by ATMs and adipocytes [86]. Interestingly, it can be a prognostic factor in various types of cancer. It was demonstrated that PAI-1 increased metastasis via induction of the mesenchymal-amoeboid transition. It can also promote tumor angiogenesis, and the lack of plasminogen decreases this process. Moreover, this factor enhances the migration of invasive cell lines by filopodia formation [101], which are one of the types of membrane protrusions, important for physical and pathological cellular processes [236].

The use of PAI as a prognostic factor was also suggested for melanoma. PAI-1 expression was detected in biopsies from patients with this cancer, in both primary and metastatic tumors. Moreover, there is a positive correlation between its level and metastasis to lymph nodes [138]. Cell lines isolated from melanoma lung metastasis had a higher level of PAI-1, which resulted in their increased invasive ability [237]. In vitro studies focusing on the suppressor functions of TGF-β on metastasis demonstrated that its downstream target is PAI-1. Induction of PAI-1 expression acts by the canonical Smad pathway and is necessary to suppress the migration and invasion of melanoma cells [234]. This study is in agreement with the previous one which showed that TGF-β decreased, in vitro and in vivo, the volume of the tumor and the migration of melanoma cells (Figure 2). This was achieved due to the reduction of plasmin production, which was a result of decreased tPA and uPA levels and elevated PAI-1 levels [238].

Moreover, this cytokine promotes melanoma growth through enhanced tumor-associated macrophages recruitment into the tumor. Macrophages with PAI-1 overexpression characterize an increased invasion rate into the melanoma spheres. Through modification of focal adhesion kinase phosphorylation PAI-1 leads to the focal contacts dissociation and motility of melanoma cells [239].

Interestingly, mice with subcutaneously inoculated melanoma cells without PAI-1 secretion after systemic administration of SK-216 (inhibitor of PAI-1) had decreased progression and angiogenesis of tumors. Therefore, the likely target of the SK-216 was the host cytokine. The authors also note that this inhibitor itself acts in an anti-angiogenic way because it has a suppressing effect on VEGF-mediated migration and tube formation of human umbilical vein endothelial cells in vitro [240]. However, other research shows that PAI-1 is an effective regulator of the invasion and formation of the blood vessels in melanoma. In PAI-1 deficient mice, angiogenesis was reduced compared to wild-type mice, while in PAI-1 overexpressing mice, angiogenesis increased. In addition, treatment of mice with low doses of PAI-1 induced tumor growth by stimulation of angiogenesis, while high doses almost completely inhibited this process and tumor growth [241]. Furthermore, administering to mice with uveal melanoma adenoviral vector encoding PAI-1 cDNA resulted in decreased frequency and severity of liver metastasis. Treated mice lived longer than control groups [242].

## 14. Conclusions

Obesity is a complex metabolic disorder that can have an impact on cancer expansion on multiple levels. Melanoma is embedded in a cell-rich niche consisting of fibroblasts, keratinocytes, immune cells, and adipocytes. All of these components are important in the development and progression of melanoma. However, under obesity conditions, fat tissue, which is composed not only of adipocytes but also of stromal cells like pericytes, endothelial, and immune cells, is the main player affecting these processes. Through the secretion of multiple bioactive molecules, adipose tissue participates in the creation of a microenvironment permissive to tumor growth and spreading. In this review, we focused on the role of AT, which possesses an altered pattern of secreted factors, in melanoma progression. This condition is associated with the de-creased production of anti-inflammatory adiponectin. Nevertheless, the secretion of the majority of hormones involved in energy metabolism is increased. Obese AT also release higher amounts of pro-inflammatory factors such as IL-6, IL-32, TNF, and chemokines. Additionally, some of the molecules like OPN, chemerin, apelin, and PAI-1, are involved in angiogenesis. All of them play a role in the regulation of melanoma growth by modifying its proliferation, migration, and invasion. We have reviewed the possible mechanisms of function of adipokines, which may act both directly or in an endocrine manner. Therefore, blockade of the adipocyte-tumor cell interactions should be taken into consideration as a promising target in the development of new therapeutic strategies. In addition, to the elements of the melanoma microenvironment, adipokines are released by the tumor cells themselves. This also stimulates the cancer progression and cross-talk between adipocytes and melanoma. Thus, understanding the function of these biological molecules will help to determine whether they may serve as predictive and/or prognostic biomarkers of melanoma progression.

## Figures and Tables

**Table 1:** Comparison of the influence of factors released by adipose tissue on physiological processes. Abbreviations: AT—adipose tissue, SAT—subcutaneous adipose tissue, VAT—visceral adipose tissue, WAT—white adipose tissue, BAT—brown adipose tissue, ↑ higher level, ↓ lower level.

Molecule	Expression in Adipose Tissue Cell Type	Type of Adipose Tissue		Level under Obesity	Main Biological Functions
Leptin	preadipocytes, adipocytes [40]	SAT [39], VAT [6]	WAT [40], BAT [38]	↑ in serum [39]	control food intake, regulation of energy expenditure, thermogenesis, inflammation, immune responses [44], regulation of bone metabolism and vascular functions [37]
Osteopontin	adipocytes [45]	VAT [46], SAT [47]	no data	↑ in serum [47,48,49] in VAT [48] ↑in SAT [47]	functions in immunity inflammation [50], control of biomineralization, calcification and bone destruction, insulin resistance [49], promotion of angiogenesis [51], induction of neovascularization [52]
Adiponectin	adipocytes [53]	VAT, SAT [53]	WAT [6,53]	↓ in serum [54,55] ↓ in SAT [56]	enhancement of glucose uptake and fatty acid oxidation, act as proangiogenic and anti-apoptotic factor in vascular endothelium [57], insulin sensitivity, act as an anti-inflammatory factor [58]
Nesfatin-1	adipocytes [59]	SAT, VAT [59]	BAT [60]	↑ in serum [59,61]	regulation of food uptake [62], control body core temperature and energy homeostasis [63], regulation of, reproduction, depressive behavior, cardiovascular and digestive systems [64], promotion of differentiation of primary brown from white adipocytes [60]
Chemerin	adipocytes [62,65]	SAT, VAT [66]	WAT [62,65], BAT [67]	↑ in serum [66]	implication in osteoclastogenesis and insulin-stimulated glucose homeostasis [68], regulation of adipogenesis, angiogenesis and inflammation [69], adipocyte differentiation [62]
CCL5	adipocytes [70]	SAT, VAT [71]	WAT [70]	↑ in serum [72,73] ↑ in SAT ↑ in VAT [71,73]	act as proinflammatory and potent anti-microbial factor [74]
Apelin	adipocytes [70,75]	VAT [76]	WAT [70,75], BAT [77]	↑ in serum [62,75]	regulation of body fluid homeostasis, angiogenesis, and energy metabolism [75], remodeling of cardiac tissue, regulation of food and fluid intake, control of the release of insulin and histamine [62]
Resistin	immune cells [55,78], preadipocytes, adipocytes [3]	SAT [56], VAT [79]	WAT [3,55,78]	↑ in SAT [56] ↑ in VAT ↑ in serum [79]	energy homeostasis [62], stimulation of inflammation, insulin resistance, enhancement a proliferation and migration of human endothelial cells and vascular smooth muscle cells [80]
CCL2	immune cells [6,72], adipocytes [72]	SAT, VAT [6,72,81]	WAT [81]	↑ in serum [72,73] ↑ in SAT ↑ in VAT [71,73]	act as proinflammatory factor [74], major chemoattractant for monocytes, NK cells, memory T cells, eosinophils and DCs [82]
IL-32	immune cells, adipocytes [83]	SAT [83], VAT [84]	no data	↑ in serum ↑ in AT [83,85]	defense against pathogens in viral infections, support chronic infection, regulation of lipid transport and metabolism, control adhesion, migration, and angiogenesis [83]
IL-6	immune cells [86], adipocytes [87]	SAT, VAT [6,72]	no data	↑ in serum [71,72] ↑ in AT [72]	differentiation of B lymphocytes into plasma cells [88], control Th17/regulatory T cells balance [89], regulate of insulin sensitivity [72], enhancement angiogenesis [90]
LIF	immune cells [91], preadipocytes [92]	SAT, VAT [6]	no data	no data	suppress food intake and body weight [93], enhancement the proliferation of hematopoietic stem cells, development and regeneration of tissues and organs, regulation immune response and inflammation [94]
TNFα	immune cells [86,95], adipocytes [81,95]	SAT [95], VAT [81,86,95]	no data	↑ in AT [72]	necessary for proliferation of cells during hematopoiesis and protection against infections [96], essential for immune regulation and morphogenesis [97], role in inflammation and angiogenesis [98], involvement in insulin resistance [72], promotion of tissue repair and of B cells differentiation [98]
PAI-1	immune cells, adipocytes [72,86]	SAT, VAT [72,86]	WAT [70]	↑ in serum ↑ In VAT [99] ↑ SAT [99,100]	main physiologic inhibitor of fibrinolysis (specifically t-PA and u-PA), enhancement of inflammation, coagulation, fibrosis, and adhesion [101], control angiogenesis and wound healing [86]
Visfatin	immune cells [102]	VAT [103,104], SAT [105]	WAT [102]	↑ in plasma [106]	regulation of cellular energetics via rate-limiting of biosynthesis of NAD, insulin-like functions, immune cell signaling [3], role in the maturation of B cells and vascular smooth muscle cells [62], promotion of migration and formation of blood vessels [55]

**Table 2:** Comparison of the adipokines expression in melanoma. Abbreviations: + detected expression, ↑ overexpression, ↓ decreased expression.

Molecule	Expression in Melanoma	Level under Obesity	Importance in Melanoma
Leptin	↑ [109]	↑ [39]	enhanced level increases the melanoma risk [111], positive correlation between serum leptin level and melanoma metastases to sentinel lymph nodes [113],
Resistin	no data	↑ [47,48,49]	no data
Visfatin	↑ [114,115]	↓ [54,55,56]	enable a distinction of melanoma from nevi or normal skin [116,117], patients with higher levels live shorter lives [115,118], positive correlation with markers of tumor mass [118] elevated expression in vertical growth phase melanoma and in metastases [114]
Osteopontin	↑ [119,120]	↑ [59,61]	higher expression in malignant than primary melanoma [121] negative correlation with patient survival and clinical outcomes in primary melanoma patients [122], prognostic marker of survival, the risk of recurrence and lymph node metastases [123], positive correlation with melanoma stage (tumor thickness, Clark’s level and mitotic index) [122,123,124,125], prognostic marker of metastatic-free and overall survival [126], positive correlation with metastases [127] into the liver [128], higher levels in invasive and metastatic melanoma compared to benign and dysplastic moles [120],
Adiponectin	no data	↑ [66]	low serum level may promote growth and more aggressive clinical course of uveal melanoma [129], low serum level improves the metastatic potential of the uveal melanoma with monosomy-3 [130]
Nesfatin-1	no data	↑ [71,72,73]	no data
Chemerin	↓ [131]	↑ [62,75]	high expression in melanoma correlates with enhanced outcome [131]
Apelin	+ [132]	↑ [56,79]	no data
CCL2	no data	↑ [71,72,73]	no data
CCL5	+ [133]	↑ [83,85]	no data
IL-32	+ [134]	↑ [71,72]	no data
IL-6	+ [119,135]	no data	higher serum level is connected with shorter overall patients’ survival [136]
LIF	↑ [137]	↑ [72]	elevated expression in melanoma with lymph node metastasis [138]
TNFα	+ [119,133,135]	↑ [99,100]	no data
PAI-1	↑ [138]	↑ [106]	no data

## Data Availability

Data sharing not applicable.
